# Widespread A-to-I RNA Editing of Alu-Containing mRNAs in the Human Transcriptome

**DOI:** 10.1371/journal.pbio.0020391

**Published:** 2004-11-09

**Authors:** Alekos Athanasiadis, Alexander Rich, Stefan Maas

**Affiliations:** **1**Department of Biological Sciences, Lehigh UniversityBethlehem, PennsylvaniaUnited States of America; **2**Department of Biology, Massachusetts Institute of TechnologyCambridge, MassachusettsUnited States of America

## Abstract

RNA editing by adenosine deamination generates RNA and protein diversity through the posttranscriptional modification of single nucleotides in RNA sequences. Few mammalian A-to-I edited genes have been identified despite evidence that many more should exist. Here we identify intramolecular pairs of Alu elements as a major target for editing in the human transcriptome. An experimental demonstration in 43 genes was extended by a broader computational analysis of more than 100,000 human mRNAs. We find that 1,445 human mRNAs (1.4%) are subject to RNA editing at more than 14,500 sites, and our data further suggest that the vast majority of pre-mRNAs (greater than 85%) are targeted in introns by the editing machinery. The editing levels of Alu-containing mRNAs correlate with distance and homology between inverted repeats and vary in different tissues. Alu-mediated RNA duplexes targeted by RNA editing are formed intramolecularly, whereas editing due to intermolecular base-pairing appears to be negligible. We present evidence that these editing events can lead to the posttranscriptional creation or elimination of splice signals affecting alternatively spliced Alu-derived exons. The analysis suggests that modification of repetitive elements is a predominant activity for RNA editing with significant implications for cellular gene expression.

## Abstract 2

A computational analysis of human RNA has identified 1,445 transcripts are edited mainly within non-coding Alu repeats, with the potential effect of regulating alternative splicing

## Introduction

On the molecular level, the complexity of higher organisms is based on the number of different gene products available for structural, enzymatic, and regulatory functions. Posttranscriptional and/or posttranslational mechanisms have an important role in generating RNA and protein diversity (Baltimore 2001). One posttranscriptional processing pathway present in higher eukaryotes is RNA editing by adenosine deamination involving modification of individual adenosine bases to inosine in RNA by adenosine deaminase acting on RNA (ADARs; reviewed in Bass 2002; Schaub and Keller 2002; Maas et al. 2003). Since inosine acts as guanosine during translation, A-to-I conversion in coding sequences leads to amino acid changes and often entails changes in protein function (Seeburg et al. 1998; Bass 2002; Schmauss and Howe 2002). The power of RNA editing in generating protein diversity lies in the fact that usually both the edited and unedited versions of the RNA and/or protein coexist in the same cell, and the ratio between the unedited and multiple edited variants can be regulated in a cell type-specific or time-dependent manner. Crucial functional properties of neurotransmitter receptors are regulated by A-to-I editing in the central nervous system (Seeburg et al. 1998; Schmauss and Howe 2002), and inactivation of editing enzymes in mice (Higuchi et al. 2000) and in the fruit fly (Palladino et al. 2000) have resulted in profound neurological phenotypes. In addition to amino acid changes, A-to-I RNA editing can theoretically lead to the alteration of transcriptional start and stop codons, as well as that of RNA splice sites. In only one case though has the creation of a splice acceptor site through intronic RNA editing been described (Rueter et al. 1999).

Currently it is not known if the recoding of mRNAs at single codon positions is the main function of A-to-I RNA editing or if other types of editing events with as yet unknown roles in the regulation of gene expression are more widespread. The recently reported embryonic lethality in mice with ADAR1 deficiency indicates that additional substrates for this enzyme exist that function during early embryonic development (Wang et al. 2000, 2004; Hartner et al. 2004). Furthermore, a role for ADAR1 in the immune system is widely accepted, as one of its isoforms is interferon induced (Patterson and Samuel 1995) and upregulated in immune cells during chronic inflammation (Yang et al. 2003). The ablation of editing enzymes in Caenorhabditis elegans resulted in transgene silencing, suggesting that the RNA editing and RNA interference (RNAi) pathways intersect (Knight and Bass 2002). This notion was recently confirmed by findings that the behavioral phenotype of ADAR-deficient worms could be rescued by inactivation of the RNAi pathway (Tonkin and Bass 2003). Since both RNAi and RNA editing target double-stranded RNA (dsRNA) molecules, RNA editing could suppress gene silencing by preventing the formation of small interfering RNAs (siRNAs).

A recurring theme of edited sequences is the involvement of an imperfectly dsRNA foldback structure (Higuchi et al. 1993). The importance of base-paired RNA elements for site-selective editing to occur is also mirrored in the presence of dsRNA binding domains in ADAR enzymes (Bass 2002). At present, though, it is not possible to predict if and to what extent a given RNA molecule is a substrate for A-to-I RNA editing in vivo.

Despite recent progress in identifying additional genes that undergo RNA editing (Morse and Bass 1997; Morse et al. 2002; Hoopengardner et al. 2003), the total number of currently known A-to-I edited genes in mammals is still small (Bass 2002). However, the activity of the mammalian editing machinery, as measured by inosine content in mRNA fractions (Paul and Bass 1998), is much higher than expected based on the current number of known substrates. Furthermore, ADARs are ubiquitously expressed in mammalian tissues, but almost all ADAR targets identified to date reside in the brain (Bass 2002; Maas et al. 2003). This discrepancy between signs that A-to-I editing is omnipresent and the scarcity of identified targets has puzzled researchers in the field for some time, wondering where all the edited transcripts are.

In this study we identify a minimum of 1,445 edited human mRNAs present in existing databases. Clusters of adenosine-to-guanosine (AtoG) discrepancies in these cDNAs are the result of RNA editing involving intramolecular pairs of inverted Alu repeat sequences, repetitive elements that represent approximately 10% of the human genome and are concentrated in and around genes (Batzer and Deininger 2002).

We also characterize functional consequences of the observed editing events and the factors that determine editing levels in Alu repeats and their modification patterns. The prevalence of Alu elements in primate genes, together with our experimental and computational analysis, suggests that the vast majority of primary human gene transcripts (greater than 85% of RNAs with average structure) are subject to A-to-I RNA editing. We show how editing might influence the alternative splicing of exonized Alu elements and discuss the implications of this extensive modification of mRNAs bearing repetitive elements for the regulation of gene expression.

## Results/Discussion

### Clusters of AtoG Discrepancies between Genomic and cDNA Sequences Are Due to A-to-I RNA Editing and They Are Located in Alu Repeat Elements

A hallmark of an A-to-I RNA editing event is an AtoG transition when comparing genomic and cDNA sequences of the affected gene since inosine base-pairs with cytosine and therefore is replaced by guanosine during reverse transcription and PCR amplification. However, AtoG discrepancies between genomic and cDNA sequences can also be due to single-nucleotide polymorphisms (SNPs) or errors in databases. Therefore the search for edited sequences on a genome-wide basis is not feasible solely based on this single feature. However, in some cases of editing, not a single, but a cluster of AtoG discrepancies between genomic and cDNA sequences is evident within a stretch of a few hundred nucleotides (Patton et al. 1997; Morse et al. 2002; Rosenthal and Bezanilla 2002). Therefore, we decided to inquire whether clusters of AtoG transitions seen in cDNA/genomic DNA (gDNA) sequence comparisons might represent bona fide editing events, since multiple base changes, all being of the AtoG type, are not likely accounted for by cosegregating SNPs or sequencing errors.

In an initial screen for candidate genes, we used the Human Unidentified Gene-Encoded (HUGE) database of ca. 3,000 human cDNAs derived from the Kazusa cDNA sequencing project (Kikuno et al. 2002). Several examples of cDNA sequences were found that within a window of 200–300 nt differ at several positions from the genomic sequence, such that the cDNA harbors a G where the genomic counterpart specifies an A. AtoG differences that coincide with an annotated A/G SNP were filtered out. Table 1 shows a list of all 26 genes from the HUGE database showing greater than two AtoG transitions in the exonic regions. Remarkably, we found that in all cases except one (KIAA0001) the location of the AtoG cluster coincides with the position of an Alu repeat element in the cDNA. As with Alu elements, most AtoG transition clusters are localized in 5′-UTR and 3′-UTR sequences and few in coding regions.

**Table 1:**
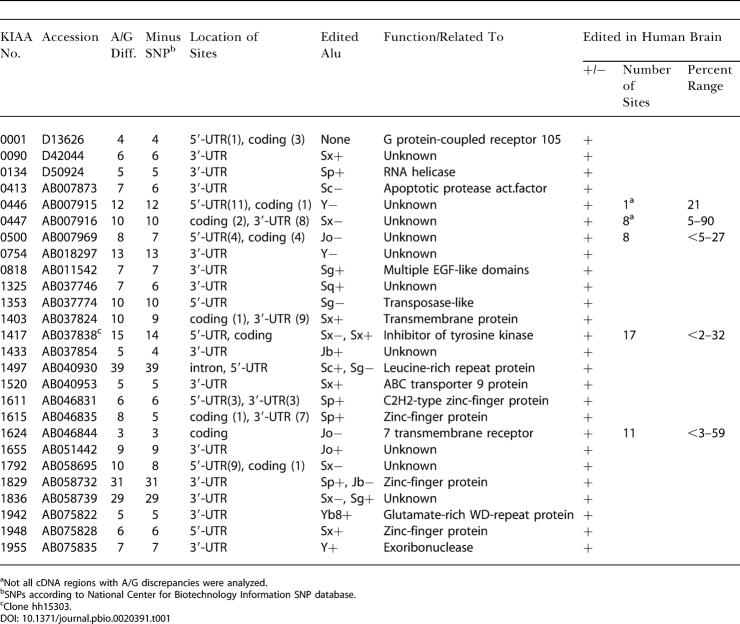
A/G Discrepancy Clusters in Human Brain cDNAs

^a^Not all cDNA regions with A/G discrepancies were analyzed

^b^SNPs according to National Center for Biotechnology Information SNP database

^c^Clone hh15303

Alu elements are short interspersed elements found in all primates, which are approximately 300 nt in length (Batzer and Deininger 2002). There are about 1.4 million copies of Alus from several closely related subfamilies present in the human genome, comprising approximately 10% of its mass (Lander et al. 2001). The enrichment of Alu repeats in gene-rich regions of the genome (Chen et al. 2002) makes their prevalence in transcribed sequences even more pronounced. Their high CpG dinucleotide content renders Alu sequences targets for methylation and implicates them in the regulation of gene expression (Rubin et al. 1994). Clusters of A/G discrepancies that mapped to Alu repeats had been noted before in the HUGE database (Kikuno et al. 2002). Furthermore, of ten newly identified editing targets in C. elegans (Morse and Bass 1999; Morse et al. 2002) and 19 in human brain (Morse et al. 2002), most were located in repeat elements. These findings suggested that repetitive elements, such as Alus, might be frequent targets for A-to-I RNA editing.

**Figure 1:**
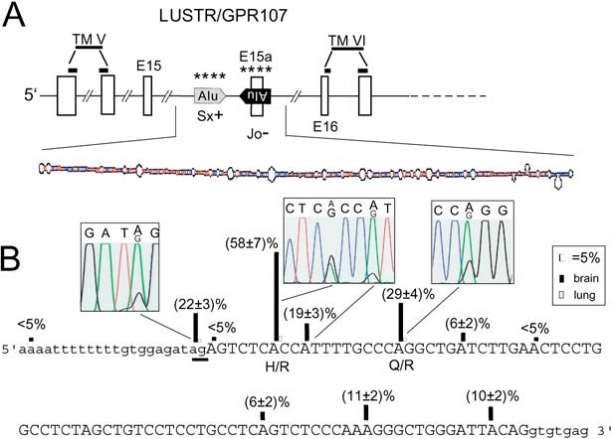
RNA Editing of an Alternatively Spliced Alu-Exon in a G-Protein Coupled Receptor (A) Schematic representation of LUSTR (GPR107, KIAA1624) gene structure around edited exon 15a. The AluSx repeat element in intron 15 and the exonic, inversely oriented AluJo are predicted to form an intramolecular foldback structure as depicted below (MFold software). TM, exonic regions predicted to encode transmembrane domains; *, editing sites. (B) Editing analysis of exon 15a (sequence in capital letters) and flanking regions. The two major editing sites predicted to change amino acids (H/R and Q/R) are indicated. Editing levels in brain (filled column) and lung (open column) are shown above each edited nucleotide. The splice acceptor site subject to editing is underlined.

**Figure 2:**
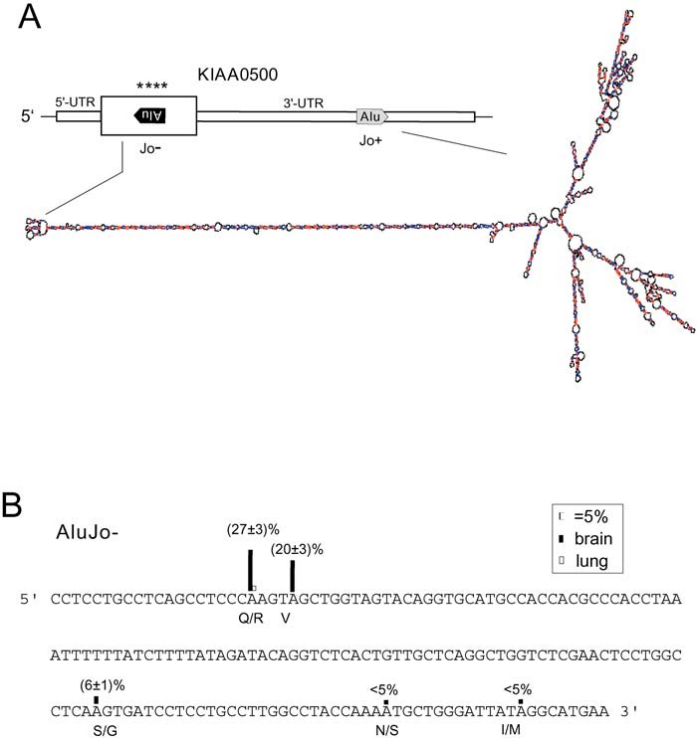
RNA Editing of KIAA500 Alu Inverted Repeat KIAA0500 is a cDNA of 6,577 nt in length cloned from human brain (AB007969) with a predicted open reading frame of 213 amino acids. Four AtoG discrepancies were present within the coding region of which two lead to an amino acid change (Q/R and S/G, respectively). (A) Structure of the KIAA500 mRNA with location of Alu elements indicated and the predicted RNA secondary structure according to the MFOLD algorithm. Large open box indicates predicted open reading frame. *, editing sites. (B) Editing analysis of an exonic Alu element in KIAA0500. Editing sites predicted to change amino acids are indicated. Our analysis revealed a significant percentage of editing (%G) at the nucleotide positions 3518 (27% ± 3%), 3522 (20% ± 3%) and 3625 (6% ± 1%) and additional editing sites with less than 5% editing, whereas parallel analysis of human gDNA confirmed the presence of adenosine at these positions. Editing levels in brain (filled column) and lung (open column, where detectable) are shown above each edited nucleotide.

**Figure 3:**
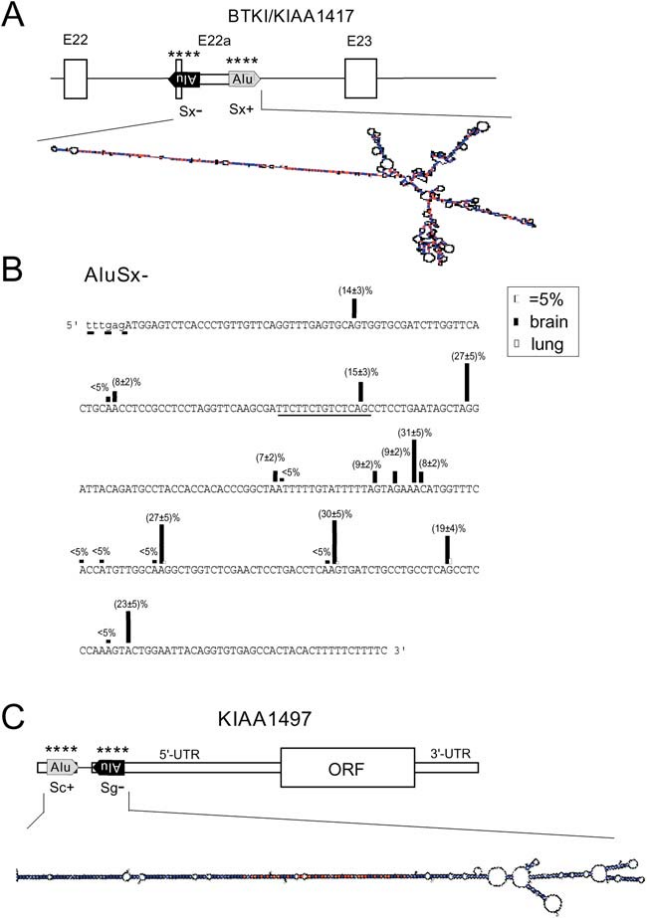
RNA Editing of Alternative Exon 22a in Inhibitor BTKI and the 5′-UTR of KIAA1497 (A) The alternatively spliced exon 22a and surrounding region of the BTKI (KIAA1417) gene with two Alu elements and its computer-predicted foldback structure. (B) Editing analysis of the AluSx- element with the exonic sequence in capital letters and edited A's in bold. The alternative splice acceptor site is underlined with a dashed line; the additional alternative consensus splice acceptor site, which undergoes editing, is underlined with a solid line. (C) Gene architecture and Alu foldback structure of KIAA1497. The brain-derived cDNA of KIAA1497, also known as LRRN1; (Taguchi et al. 1996), has a total of 15 nonpolymorphic AtoG discrepancies to gDNA, 14 being located within the 5′-UTR of the gene and one within the coding region. We analyzed PCR products covering all 14 potential editing sites in the 5′-UTR for editing in cDNA from human brain and could confirm in vivo editing to an extend clearly above the detection limit of our method for most of these positions and also at additional adenosines (data not shown). ORF, open reading frame; *, editing sites.

In order to better understand the connection of Alu's with the observed AtoG clusters, we analyzed experimentally the cDNAs from all 25 candidate genes for RNA editing in human brain. Total RNA and gDNA were isolated from the same human brain specimen to eliminate false positives from unmapped A/G SNPs. For all 25 genes in vivo RNA editing was detected by single-run sequencing of gene-specific RT-PCR products, and for five of them the editing efficiency was quantitatively evaluated through repeated experiments. Extents of editing ranged from less than 2% to 90% at individual sites (Table 1; Figures 1–3).

### Intramolecular Pairs of Oppositely Oriented Alus Are Responsible for Alu Element Editing

**Figure 4:**
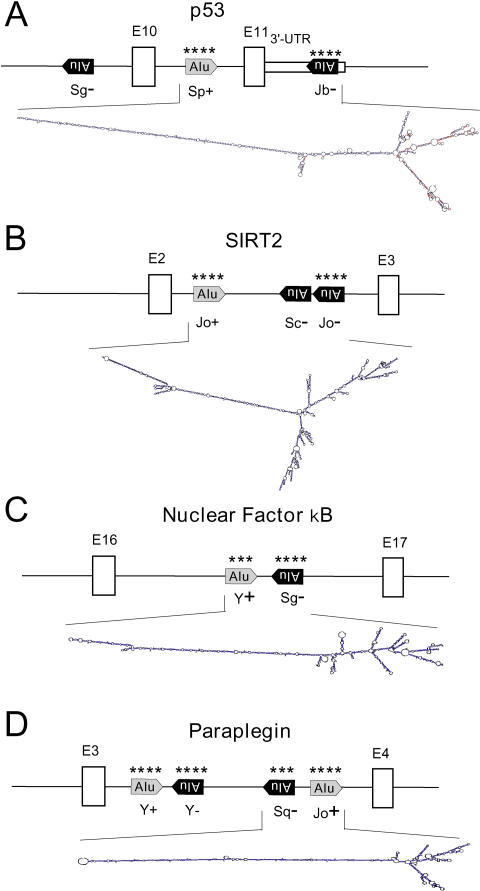
Alu-Mediated RNA Editing in p53, SIRT2, NFκB, and Paraplegin Pre-mRNAs Schematic presentation of the gene structures from (A) P53, (B) SIRT2, (C) NFκB, and (D) SPG7. Edited repeat elements are marked by asterisks. RNA folds appear as calculated with MFOLD. The AluJb− in p53 is located in the 3′-UTR (A); all others are intronic. *, editing sites.

Since a prerequisite for A-to-I RNA editing is the presence of a partially base-paired RNA foldback structure (Higuchi et al. 1993; Bass 2002), the observed modifications in Alu repeats might be the result of two oppositely oriented, base-pairing repeat elements located within the same RNA molecule. For each of the 25 genes with edited, exonic Alu elements we find such oppositely oriented Alu repeats in the same pre-mRNA, many of which are located in intronic sequences. To determine if the predicted Alu pairs and the calculated foldback structures (Figures 1A, 3, and 4) actually form in vivo, we analyzed experimentally the predicted Alu partners from the pre-mRNA for four of the identified editing targets (LUSTR, KIAA0500, Bruton's tyrosine kinase [BTKI], and KIAA1497). In each case we found that the closest, oppositely oriented Alu repeat undergoes A-to-I RNA editing as well.

Because of the abundance of Alu elements in human pre-mRNAs, most primary transcripts contain one or more pairs of oppositely oriented Alus. If a majority of them is indeed subject to A-to-I RNA editing in vivo, it should be possible to predict RNA edited genes by identifying inverted pairs of Alu repeats in pre-mRNA transcripts. As a proof of principle, the analysis was extended to four arbitrary chosen genes (p53, SIRT2, NFκB, and paraplegin (SPG7) containing pairs of Alu repeats as seen schematically in Figure 4B–4E. In all four cases, editing in the Alu elements that are predicted to form a dsRNA foldback structure is readily detectable.

**Figure 5:**
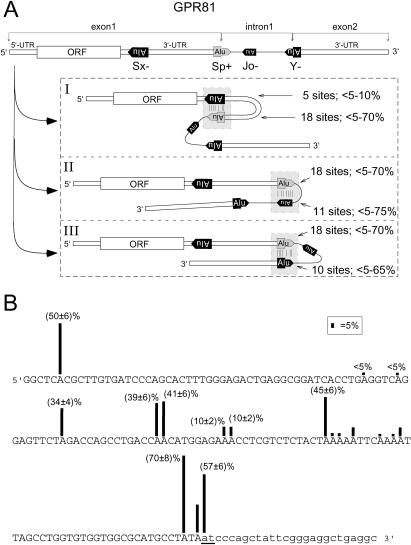
Editing of Alternative Foldback Structures of GPR81 Pre-mRNA (A) The position and orientation of all four Alu elements in GPR81 pre-mRNA is indicated. Three alternative Alu pairings (I–III) are predicted and experimental editing analysis indicates that all three do form in vivo. ORF, open reading frame; *, editing sites. (B) Editing analysis of AluSp+ in GPR81. Percentages of editing in human brain are indicated. The exonic sequence appears in capitals. The edited AT dinucleotide that becomes a splice donor site is underlined.

Many primary gene transcripts allow several energetically favorable foldback structures to be predicted for a given Alu that involve different combinations of Alu pairs. Do all these alternative Alu-pair foldback structures exist in vivo and are therefore subject to RNA editing? To address this question we examined the editing pattern of the G-protein-coupled receptor 81 (GPR81; identified through a computational search as described below). GPR81 contains four Alu elements, one sense and three antisense oriented, in the 3.6-kb pre-mRNA and was selected based on Alu repeat configuration and transcript length. If the alternative foldback structures depicted in Figure 5 coexist in vivo, all four Alu elements should show signs of editing with the level of editing indicating how prominent each of the alternative structures is. According to the analysis of GPR81 pre-mRNA, all three configurations form in vivo with variant II possibly being the dominant one since AluSp and AluJo show the highest levels of editing (Figure 5).

These results suggest that Alu elements in human mRNAs are subject to RNA editing by ADARs because of foldback structures formed between two oppositely oriented Alus present within the same primary transcript.

### Editing of Alus Is Tissue Dependent and It Alters Codons and Pre-mRNA Splice Sites of Alternatively Spliced Alu Exons

Exonic Alu repeat elements are predominantly located in the 5′- and 3′-UTRs of mRNAs, and as a result, most cases of Alu editing occur in noncoding regions. However, some editing events predict amino acid changes (Table 1). Among the identified genes for which we performed a detailed, quantitative editing analysis several unique and recurring features emerge regarding the locations and functional implications of the editing events.

The LUSTR1 cDNA codes for a G-protein-coupled seven-transmembrane receptor (also termed GPR107 or KIAA1624), with three AtoG discrepancies located within an alternatively spliced AluJo-derived exon that leads to the in-frame insertion of 29 amino acids between transmembrane regions V and VI of the protein (see Figure 1A). The experimental analysis revealed a total of ten editing sites within this Alu element (see Figure 1B), including two major sites that lead to amino acid changes (H/R and Q/R sites). Interestingly, editing levels at all positions were significantly different in human brain (19%–58%) compared to lung (less than 5%), suggesting a tissue-specific regulation of editing (see Figure 1B).

Analyzing the RNA editing pattern of LUSTR1 pre-mRNA revealed additional intronic editing sites, one of which represents the splice acceptor adenosine (AG to IG) in intron 15 (22% edited in brain; see Figure 1B). Editing at this position is predicted to lead to the exclusion of the Alu exon, indicating that the alternative splicing of exon 15a might be coregulated by RNA editing of its splice junction. This is to our knowledge the first documented example where A-to-I RNA editing acts to destroy a pre-mRNA splice signal.

A picture similar to LUSTR1 emerges from analysis of the gene for human inhibitor of BTKI (also termed KIAA1417; Liu et al. 2001; Strausberg et al. 2002). Again, an alternatively spliced Alu exon (located between constitutive exons 22 and 23) is affected. This time two AluSx elements are positioned in opposite orientation at the start and end of the exon (see Figure 3). Inclusion of the exon using the splice acceptor site provided by AluSx− leads to the premature termination of translation within this exon with all editing sites located in the 3′-UTR. Editing levels at 20 sites throughout the Alu element range from less than 5% to 31% in human brain, whereas cDNAs isolated from human lung again displayed few editing sites with low editing levels of less than 5%. A splice site is also subject to editing in BTKI, this time affecting an additional alternative splice acceptor site within AluSx−. On the pre-mRNA level this position is edited to 15%. However, in transcripts that use the weak upstream splice acceptor site (underlined with a dashed line in Figure 3B; as in the HUGE database clone hh15303), the additional alternative splice site (underlined with a solid line in Figure 3B) is highly edited, raising the possibility that edited BTKI pre-mRNA preferentially follows the alternative splicing pathway (data not shown).

The analysis of GPR81 revealed another case of Alu exon alternative splicing and, surprisingly, a new mechanism showing how RNA editing might affect RNA processing. Within the AluSp+ element located in the 3′-UTR of GPR81 transcripts a splice donor site (AT to IT) is generated in 57% of primary transcripts by RNA editing. This is predicted to give rise to alternatively spliced mRNA products represented by GenBank entry AF385431 (see Figure 5B). This is, to our knowledge the first reported case of potential splice donor site creation by RNA editing. It is possible that here the Alu element was inserted into the 3′-UTR exon of the GPR81 gene and has evolved into a state where it is a single mutation away from initiating the birth of a novel intron. Posttranscriptionally RNA editing provides the final base change to create the new splice site. This scenario is supported by the fact that in mice the GPR81 gene lacks introns.

It is intriguing that we find cases where editing in alternatively spliced Alu exons, or within adjacent splice sites, interferes with or counteracts exon formation of an Alu repeat. It suggests that RNA editing might be more generally involved in the regulation of Alu exonization. Recently, it has been shown that more than 5% of the alternatively spliced exons in the human genome are Alu derived (Sorek et al. 2002). Exonization of Alu repeats occurs via activating mutations in mostly antisense-oriented, intronic Alus generating a novel splice acceptor site (Mitchell et al. 1991; Lev-Maor et al. 2003), and it has been speculated that exonization of transposable elements in general is a major mechanism for the generation of novel exons (Kreahling and Graveley 2004). A large number of intronic Alu elements are just a single mutation away from being exonized (Lev-Maor et al. 2003; Kreahling and Graveley 2004), and in some cases the constitutive splicing of an intronic Alu has been shown to cause a genetic disorder (Mitchell et al. 1991; Knebelmann et al. 1995; Vervoort et al. 1998). In this context RNA editing may partially counteract genomic mutations that lead to the incorporation of deleterious novel exons while maintaining their potential to form exons with beneficial functions through further mutation.

Furthermore, RNA editing in Alus might be involved also in the generation of novel introns as seems to be the case in GPR81. Statistically, however, the exonization of intronic Alus would be much more frequent than the intronization of exonic Alus because of the abundance of Alus in introns.

### A Transcriptome Wide Screen for Edited Alu Repeats

The results presented above show that clusters of AtoG mismatches in cDNA/gDNA sequence comparisons represent an effective way to identify authentic editing cases with a low rate of false positives. Since all clusters of AtoG discrepancies mapped to repeat elements, we wondered how prevalent the editing of Alu or other repeat elements is in the human transcriptome. Therefore, we devised a database search procedure to identify pairs of inverted repetitive elements in human mRNAs exhibiting AtoG transitions.

Initially, a limited search was carried out for closely spaced (less than 2 kb) inverted pairs of human Alu, MIR, and L1 repeat elements that overlap with exonic sequences and for which an mRNA sequence can be found in GenBank entries. This search, involving about one-third of all repeat elements in the human genome, identified 71 mRNAs with exonic repetitive-element pairs (51 Alu, six L1, six MER, and eight MIR). From those mRNAs, 27 displayed clusters of AtoG changes, all in Alu elements. Fourteen of these genes were chosen for experimental analysis, and all 14 proved to be subject to A-to-I RNA editing (Table 2). Since these initial results indicated a high prevalence of editing in Alu elements, we decided to carry out a comprehensive search involving all elements present in cDNA sequences.

**Table 2:**
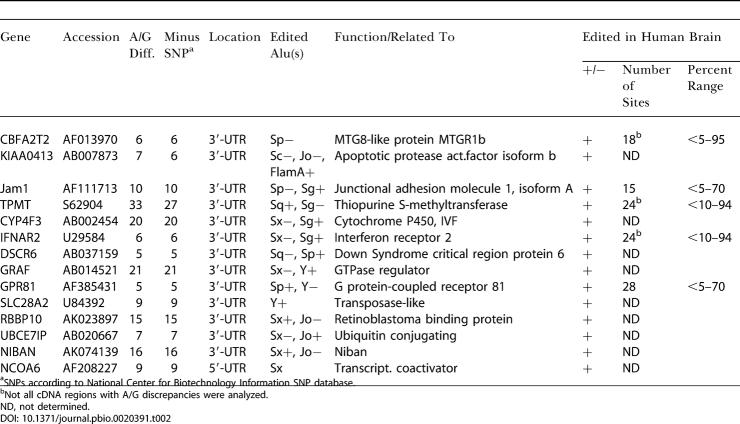
Edited Alu Exons from Computational Screen for Alu Element Foldback Structures

^a^SNPs according to National Center for Biotechnology Information SNP database

^b^Not all cDNA regions with A/G discrepancies were analyzed

ND, not determined

**Figure 6:**
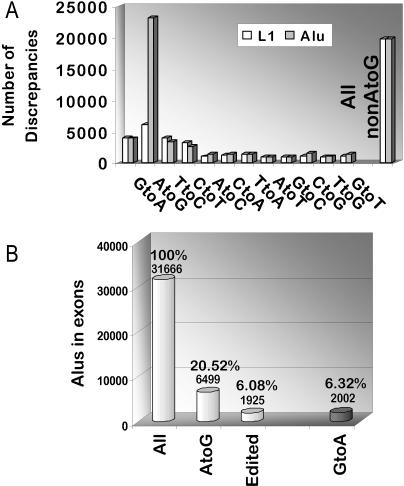
Mismatch Bias in Exonic Repetitive-Element Sequences (A) Plot of the nature and number of mismatches within Alu and L1 sequences present in human cDNAs. For reasons of comparison the L1 mismatch numbers have been multiplied by 2.9 so that the non-AtoG mismatch count for Alu and L1 is identical. Transition mismatches AtoG, GtoA, CtoT, and TtoC are displayed together for comparison. (B) Plotted are the total number of Alu sequences found in human cDNAs (first column) and the number of elements harboring AtoG and GtoA mismatches (second and last column). The third column indicates the high confidence set of edited elements (α = 0.000001).

We analyzed the total of 103,723 human mRNA sequences (from the University of California, Santa Cruz [UCSC] Genome database [Kent et al. 2002], July 2003 assembly) for overlaps with repetitive elements of the L1, Alu, MaLR, and MIR families. For Alus, 17,406 mRNAs (16.8%) contained a total of 31,666 complete or partial repetitive-element sequences. Comparing the cDNA sequences with their genomic counterpart revealed that the number of AtoG discrepancies within Alu repeats is more than seven times higher than the average number of the other transitions (23,204 versus 3,271 [the average for GtoA, CtoT, and TtoC transitions]). In fact, the number of AtoG mismatches is higher than all other eleven types of nucleotide discrepancies combined (Figure 6A).

While the finding that non-AtoG transitions (GtoA, CtoT, and TtoC) are approximately three times more frequent than transversions is in line with results from previous studies analyzing gDNA sequences (Lander et al. 2001; Venter et al. 2001), there is no explanation for the observed excess of AtoG mismatches relative to other base transitions. Alu sequences carry 22–23 CpG dinucleotides, which are known to show high mutation rates because of C-methylation, and as a consequence, these positions should display an elevated frequency of SNPs. Nevertheless, an elevated number of SNPs would lead to a rise in AtoG as well as GtoA mismatches when comparing a representative population of cDNAs with the corresponding genomic sequence. Thus, we concluded that the excess exclusively in the number of AtoG discrepancies in Alus over other base changes may reflect cases of bona fide A-to-I editing at the RNA level.

We then devised a statistical approach to distinguish repetitive elements that show AtoG mismatches due to sequencing errors and SNPs from those that have undergone A-to-I RNA editing. The method was based on the observation above that Alu elements subject to RNA editing undergo multiple base modifications that result in a cluster of AtoG discrepancies (5–30) between cDNA and gDNA. The probability that a cluster of several AtoG discrepancies is due to sequencing errors or SNPs (in the absence of an increased number of other nucleotide discrepancies indicating low-quality sequence data) is negligible. Thus the number of clustered AtoG changes can be used to distinguish genuinely edited elements from elements with aberrant or non-editing-related base changes. For each Alu element with AtoG discrepancies, we computed the χ^2^ test comparing the observed number of AtoG discrepancies with the expected number, based on the number of non-AtoG mismatches present in the same sequence. Elements with a χ^2^ higher than the critical value for α = 0.00001 (corresponding to a probability of one in 100,000 that the observed AtoG transitions are due to SNPs or sequencing errors) were selected as “edited” and will be called so throughout the rest of the manuscript. Using this approach we found that out of those 17,406 mRNAs with one or more exonic Alu elements, 1,445 (8.0%) mRNAs are edited within one or more of the Alu sequences (for a full list of edited mRNAs see Table S1). When looking at all the 31,666 Alu elements present within these 17,406 RNAs, we find that 1,925 (6.1%) Alu elements are “edited,” while another 4,574 Alu elements (14.4%) show AtoG discrepancies but fail to pass our probability cutoff (Figure 6B). Thus, the total of 6,499 elements (or 20.5%) represents the upper limit of potentially edited Alus in our sample (Figure 6B). The total number of Alus with GtoA discrepancies in the same sequence sample is 2,002, and we considered this value to reflect base changes that are due to SNPs and sequencing errors. Assuming a similar number for random AtoG and GtoA mismatches, we can subtract this number from the total count of potentially edited Alus, obtaining 4,497 cases (14.2%) as the approximate number of actually edited elements.

In order to validate our screening approach, we performed an identical analysis for GtoA, CtoT, and TtoC mismatches. Compared to the 1,925 AtoG-edited Alu elements in mRNA, we found 12 GtoA, 11 CtoT, and 11 TtoC cases of “editing.” These cases may represent false positives and thus set the error level of our screen to less than 0.6%.

These results suggest that out of the 103,723 human mRNAs at least 1.4% are A-to-I edited within an exonic Alu element. Apart from Alu repeats, many more low- and high-frequency repeats exist in the human genome (Venter et al. 2001) and might give rise to RNA foldback structures that result in exonic A-to-I editing. Therefore, the total number of mRNAs edited in exonic repeat sequences is probably higher than the value obtained from our analysis of Alu elements.

Most Alu repeats are located in introns, and it is there where the bulk of RNA editing is expected to occur. The average number of Alu repeats per gene is 12.4 estimated for Chromosomes 21 and 22 (Grover et al. 2003). This value is comparable to the 19.3 Alus/gene estimated from our data (2,003,976/103,723: total number of Alus (nonunique) in mRNA boundaries/number of mRNAs) for the whole genome. Considering that based on our analysis 14.2% of exonic Alus are edited, and assuming similar editing rates for intronic Alus, we can estimate that the probability of an average pre-mRNA to be edited is approximately 1–0.858^19.3^ or 94.7% (85.0% with the 12.4 Alu/gene estimate). While this value is an approximation, assuming that all genes have similar structures, and does not take into account editing in other repeat elements, it does reflect the magnitude of repetitive-element editing.

### Distance, Conservation, and Tissue Localization Influence Which Pairs of Alu Elements Are Edited

To gain insight into the factors that determine which Alus are subject to RNA editing, and under what circumstances, the identified set of 1,925 high-confidence cases of editing in Alu elements (contained in the 1,445 mRNAs listed in Table S1) was used for further computational analysis.

**Figure 7:**
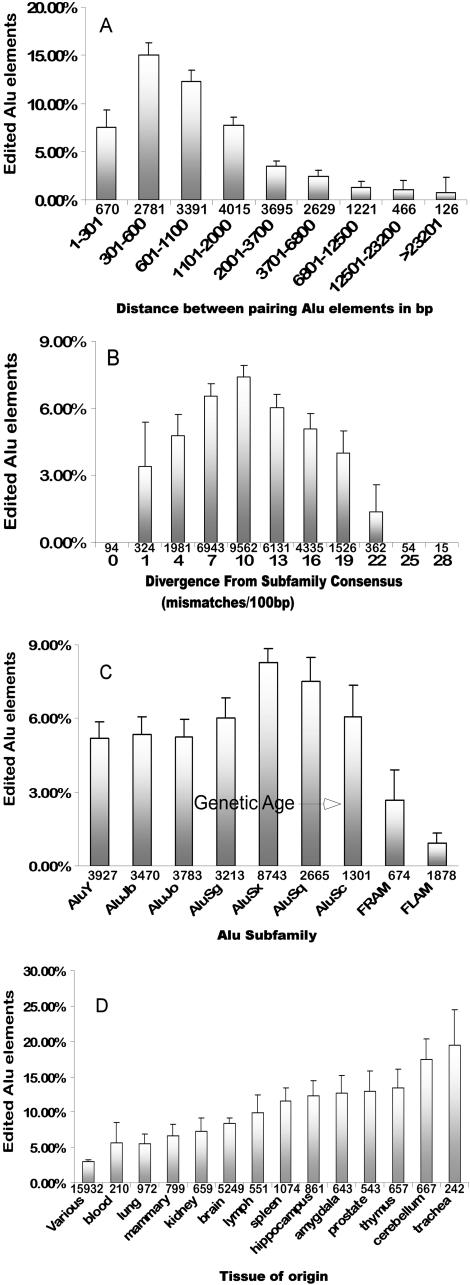
Factors Determining Alu Targeting Probability (A) Percentage of edited elements classified in bins according to the distance separating the element and its closest inverted Alu partner. (B) Percentage of edited Alu elements clustered according to divergence from their corresponding Alu-subfamily consensus. (C) Percentage of edited Alu elements in each Alu subfamily. In (A), (B), and (C) the numbers at the bottom of the bars show the sample size in each bin. (D) Percentage of edited elements according to the tissue from which the RNA was isolated. Error bars show 95% confidence levels.

It was assumed that the observed editing is the result of RNA foldback structures formed between intramolecular inverted Alu repeats, as we have demonstrated for all the experimentally analyzed cases. If this hypothesis is correct, then the distance between an Alu and its closest inverted pairing element should be a critical determinant for how likely it is that a given element will be targeted by the RNA editing machinery. To test this hypothesis the closest inverted Alu was identified within the same gene for all 31,666 Alu elements. A properly oriented element was found for 19,231 of those Alu elements, and a plot was made showing the percentage of edited Alus as a function of the distance between elements (Figure 7A). The highest level of editing (approximately 16%) was found for Alu pairs 300–400 nt apart, which corresponds to slightly more than the size of a full-length Alu repeat. Editing levels subside with increasing distance as the probability for base-pairing between the two Alu elements apparently decreases. Alu pairs with distances below 300 nt indicate partial Alu elements, and the observed decrease in editing levels is likely because of the smaller, less energetically stable foldback structures. These results suggest that the optimal configuration of an Alu-pair stem loop involves two full-length Alus forming the stem separated by a short (10–50 bp) intervening loop sequence. Interestingly, as the distance increases we ultimately arrive at a low-level plateau of approximately 1% editing without any further drop in editing levels. RNA editing in *trans* caused by base-pairing Alus located in separate RNA molecules might be responsible for this “background” editing. A-to-I editing in *trans* does occur on pre-annealed RNA duplexes in vitro (Bass and Weintraub 1987; Nishikura et al. 1991) and could occur also in vivo if such intermolecular RNA duplexes form. In *Xenopus* one case of potential *trans* editing has been described, involving RNA duplexes formed between sense and antisense transcripts of bFGF (Saccomanno and Bass 1999).

The distance dependence of the extent of editing clearly suggests that the formation of Alu–Alu stem loop structures predominantly results from intramolecular Alu inverted repeats with an upper limit of approximately 1% editing that could be due to intermolecular Alu pairings. To our knowledge, these results describe for the first time the distance relationship of long-range RNA folding interactions in vivo and how their stability is influenced by distance.

More important, considering the high frequency of Alu elements in primate RNA sequences and the low levels of potential intermolecular editing observed, we conclude that intermolecular duplexes between complementary RNA sequences do not form in the nucleus at a significant rate. This raises the question of how the regulation of thousands of human messages proposed by Yelin and colleagues involving antisense transcripts works (Yelin et al. 2003). It might also explain why cases of editing involving endogenous sense/antisense RNA duplexes have not been reported despite evidence for extensive antisense transcription.

The editing of RNAs by ADARs has been shown to be dependent on the double-stranded character of the substrates, such that editing levels and promiscuity increase with the extent of the base-paired region (Bass 2002). The human Alu family is composed of several subfamilies of different genetic ages, and their consensus sequences contain diagnostic changes distinguishing one subfamily from another. The extent of base-pairing between two oppositely oriented Alu elements, and in turn the extent of A-to-I editing, depends on their sequence homology, and it is expected that highly diverged elements would form less stable foldback structures. The relationship between observed editing level in our set of 31,666 Alu repeats and the sequence divergence of each Alu repeat from the consensus of its respective subfamily is shown in Figure 7B. A decrease in editing levels is seen with an increase in diversity, suggesting that an Alu element with lower sequence homology to most other Alu repeats has a lower probability of forming a suitable editing substrate. Unexpectedly, we also observed a drop in editing levels for Alu elements with low divergence from their subfamily consensus sequence. This trend may be caused by the distribution of Alu divergence. Within the human genome the majority of Alu elements have diverged by 10%–15% from their subfamily consensus (Stenger et al. 2001). Therefore, Alu elements with lower than average divergence have a lower likelihood of encountering another element of similar divergence, resulting in low editing levels for this subset. We obtained similar results when we compared the editing levels of Alu elements with the sum of the divergence of the edited Alu and its closest inverted Alu element (data not shown). In agreement with these conclusions, we find that the most populated Alu subfamily (AluSx) and the subfamilies closely related to AluSx sequences (AluSq, Sc, and Sg) show the highest levels of editing (Figure 7C).

The pool of mRNAs used in this study represents a heterogeneous collection of sequences from different tissues and cell types. In analyzing the editing of Alu elements as a function of tissue origin (Figure 7D), significant differences in editing levels were found. The highest editing activities were seen in brain tissues, in trachea and thymus. These results are in accordance with prior studies that have measured the overall activity of RNA editing enzymes in selected mammalian tissues as judged by the amount of inosine detectable in the poly(A)+ fraction of RNA (Paul and Bass 1998). The two human enzymes with A-to-I RNA editing activity (ADAR1 and ADAR2) display a different but overlapping activity profile on known substrates, and their expression is highest in brain (ADAR2) and in cells of the immune system (ADAR1; Bass 2002). Furthermore, ADAR1 was found to be induced during inflammation leading to high activity in blood cells and thymus (Yang et al. 2003). These findings are also in agreement with our experimental results, which show much higher editing levels in brain-derived RNAs than in the same mRNA isolated from lung tissue (see Figures 1–3).

The pool of edited Alu elements was analyzed for other features that might influence editing levels, such as the position of the edited Alu within the mRNA (3′-UTR, 5′-UTR, and coding region) or its orientation in relation to the mRNA (sense, antisense). No significant correlation of Alu editing was detected with any of these features (data not shown).

### Editing of Alu Repeats Shows Sequence and Structure Preferences

**Figure 8:**
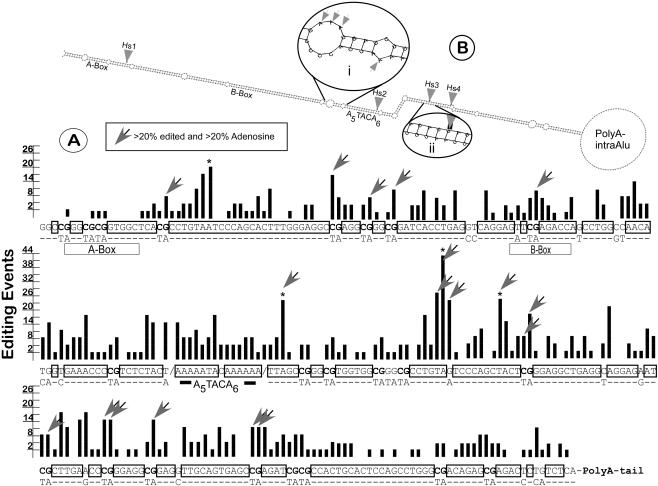
Sequence and Structure Preferences of Editing in Alus (A) The consensus sequence of 141 edited full-length Alu elements present within human Chromosome 1 transcripts with the number of editing events indicated for each sequence position (bars). Insertions and deletions present in fewer than five elements are not shown in the alignment for clarity. Bases conserved in more than 80% of the sequences are boxed. For the lesser conserved consensus positions the next most frequent base is listed below. Consensus CpG dinucleotides are in bold. Arrows indicate “high-efficiency” positions where more than 20% of adenosines present appear to be edited. Note the overlap of these positions with CpGs. Major features of Alu sequences, such as the A-Box and B-Box of Pol III and the Alu polyA sequence are labeled. (B) A typical Alu foldback structure and its major features as discussed in the text. Arrows indicate TA hot-spot positions. The magnifications show the two typical configurations of editing sites found in Alu pairs: mismatched A/C bulges (i) and A/U base pairs (ii).

The availability of such a large collection of A-to-I edited sequences resulting from this analysis allowed us to examine the modification pattern of edited Alu elements for potential editing hot spots or base preferences. To this end we first aligned all 141 edited Alu sequences (greater than 260 bp) in RNAs originating from Chromosome 1 and mapped the edited sites on their consensus sequence (Figure 8). Interestingly, certain adenosines are targeted in greater than 30% of the edited RNAs while other adenosines do not show any evidence of editing. The four editing hot spots all map to TA dinucleotides that are located in conserved Alu regions (greater than 80% identity), suggesting that they are base-paired in the average foldback structure. This confirms the previously proposed T/A 5′-neighbor preference for both ADARs (Polson and Bass 1994). Surprisingly, most of the 22 CpGs of the consensus sequence coincide with the location of high-frequency editing events. CpGs are known to be targeted by cytosine DNA methylation, which results in a high mutation rate, turning CpGs into either CA or TG dinucleotides (Batzer and Deininger 2002). Since less than 50% of the transcripts carry a CA or TG (edited in reverse complement) at these CpG consensus sites, the editing efficiency (edited adenosines/total adenosines) at these positions is comparable to that at the hot spots (Figure 8A, arrows).

As a result of the high CpG mutation rate, frequently the Alu foldback structure of the unedited RNA is predicted to carry A–C mismatches at these positions. Editing at these sites restores the CpG repeat (CA→CI) on the RNA level and converts the A–C mismatch to an I–C base pair. Energy calculations for several predicted Alu pairs show, surprisingly, that the stability of the foldback structure is not diminished by editing but often increased because of the high frequency of I/C pair formation (data not shown). It is therefore unlikely that in the case of Alu foldback structures, RNA editing serves to resolve RNA secondary structures that interfere with the processes of splicing or translation of these RNAs, as suggested previously (Morse et al. 2002). Two typical configurations of editing sites observed in Alu elements are depicted in the magnifications of Figure 8B where either A–U pairs are turned into I–U wobble pairs in conserved regions of the sequence (ii), or A–C mismatches are converted into I–C pairs within nonconserved Alu regions (i).

**Figure 9:**
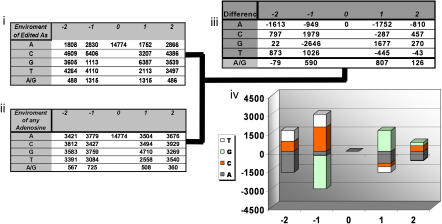
*Cis* Preferences of Editing Sites in Alus Tables (i) and (ii) show the frequency of A, G, C, T, or an A/G editing site at positions −2, −1, 1, and 2 relative to each of the 14,774 AtoG mismatch sites found within the high confidence group of Alu elements (i) and in relation to a randomly chosen adenosine from each of the those sequences for each AtoG mismatch (ii). Table (iii) shows relative editing preferences after bias removal by subtracting table (ii) from Table (i). (iv) Graphical representation of Table (iii).

While the above analysis shows the qualitative features of the editing sites in Alus, determination of *cis* preferences was carried out by extracting 14,774 pentanucleotide sequences with the edited adenosine as the middle base and estimating the frequency of each base at positions −2, −1, 1, and 2 relative to the editing site. To correct for Alu sequence bias we performed the same analysis for a randomly chosen adenosine for each edited adenosine in our sample. We then subtracted those frequencies to obtain unbiased editing preferences (Figure 9). The presence of large, unpaired poly(A)+ tails in Alus obscures our analysis for adenosines surrounding edited A's but is informative regarding other base preferences. Position −1 shows a strong preference for C and T and aversion for G in agreement with previous studies (Bass 2002). Interestingly, we observe preferences for G in position +1 and for C or G at positions −2 and +2, which have not been described before. This preference pattern appears not to be linked to any Alu-specific structural feature and therefore possibly reflects the editing enzyme *cis* preferences. We also identify a preference for an editing site to be preceded or followed by another editing site (Figure 9). This data-rich assessment of sequence preferences for edited sites might be useful in an ab initio identification of new editing sites. Taken together our results identify loose RNA duplexes carrying A–C mismatches or A/U-rich regions, as favored editing targets. The high incidence of “corrective” editing at mutated CpG consensus positions in Alus raises the possibility that posttranscriptional restoration of CpG repeats in Alu primary transcripts by RNA editing contributed to the surprising retention of CpGs in Alus during evolution (Batzer and Deininger 2002). This might constitute an important consequence of A-to-I editing in view of the role of CpG islands in the regulation of gene expression.

### Potential Functional Implications of RNA Editing in Repetitive Elements

Considering the available data on in vitro editing activities of ADARs on dsRNA molecules of different sequences and structures, it is not surprising that highly base-paired RNA foldback structures such as the ones induced by Alu inverted repeats are substrates for the editing enzymes. However, it is remarkable and maybe surprising that these predicted structures are edited in vivo at significant levels. This indicates that many of these structures do form in vivo and are readily accessible for ADARs in the nucleus.

Alu elements are ideal for the formation of editable RNA structures because of their large numbers, size, and degree of conservation. We find no evidence for a sequence or otherwise specific interaction of the editing machinery with Alu sequences. Thus, other repetitive elements able to form similar structures should also be targets of A-to-I editing. Our data suggest, however, that editing levels in all other major repeat-element families that dominate the human genome (LINE, LTR, and other short interspersed elements) are very low compared to editing levels seen in Alu repeats (see Figure 6A and unpublished data). The selectivity for Alus might be explained based on the distribution features of each repetitive-element family: For example full-length L1 repeats are approximately 6 kb in length, and as a consequence, most of the time they have low chance of having a base-pairing sequence in proximity. MIR repeats, although found in significant numbers, which potentially could form foldback structures, have a low average level of conservation (30%–40% divergence) and so may be inadequately double stranded to be a substrate. MaLR elements of the LTR superfamily are present in numbers such that the average distance between an inverted pair is very high (approximately 10 kb). However, our analysis suggests that all repetitive elements might become targets of RNA editing at different stages in evolution. Young repetitive elements in their expansionary phase of evolution display features that we identify as important for being editing targets. Based on these observations it will not be surprising if repeat elements that show low levels of editing in humans are major targets in other organisms.

For mRNA fractions, we estimated the inosine content due to Alu editing as follows: In 103,724 mRNAs we found 23,204 AtoG mismatches, while the same sequence sample has an average for the other transitions of 3,271. Assuming an average mRNA size of 4 kb, the ratio of inosine in the sample is estimated to be one inosine every 20,814 nucleotides (103,724 × 4,000/[23,204–3271]) generated by editing in Alu sequences. This estimation for Alu editing is in the range of one inosine in 17,000 nt (brain), one in 33,000 nt (lung, heart), to one inosine in 150,000 nt (skeletal muscle) as was experimentally determined by Bass and colleagues in the polyA-fraction of rat RNAs (Paul and Bass 1998). Since the rat genome lacks Alus, the total amount of inosine generated in human mRNAs may be much higher than in rats, unless a class of edited sequences in rats exists with a similar prevalence to Alus in humans. In any case, our data imply that most of the inosine detected in mRNA transcripts can be explained by the widespread A-to-I editing of repetitive elements. Repeat-element editing might therefore point toward an important housekeeping function for RNA editing. In contrast, the well-studied examples of editing that lead to single nucleotide and codon changes in mRNA might be less frequent cases of editing events.

While a significant amount of editing occurs in mRNAs that contain repetitive elements in their exons, our results predict that the bulk of A-to-I editing takes place in intronic sequences missing from cDNA databases. This is suggested by the experimental results regarding the LUSTR, GPR81, p53, SIRT2, NFκB, and paraplegin genes, for which intronic data was available (see Figures 1A, 4, and 5A). This extensive editing of repetitive elements in pre-mRNAs creates an enormous pool for the generation of gain-of-function mutations. The involvement of editing in creating or destroying splicing sites of alternatively spliced Alu exons, along with internal editing of those exons, suggests an intriguing new mechanism for accelerated evolution. We are now in a position to analyze the extent to which this process occurs within the human transcriptome. Such a role in “stimulating” evolution, however, is unlikely to be related to the “daily” function of A-to-I RNA editing.

It has been shown that hyperedited, inosine-containing RNAs are retained in the nucleus by a protein complex containing the inosine binding protein p54 (Zhang and Carmichael 2001). In view of the widespread editing of Alus this offers an intriguing mechanism to preclude aberrantly spliced mRNAs and, more generally, repetitive-element-containing RNAs from exiting the nucleus. This model, though, suggests that intronic RNA editing occurs frequently in other organisms and in other repetitive-element types as well, something that remains to be shown.

A connection between A-to-I RNA editing and RNAi has recently been suggested through studies in C. elegans where inactivation of the editing machinery leads to transgene silencing (Knight and Bass 2002), and subsequent inactivation of the RNAi pathway restored transgene expression (Tonkin and Bass 2003). Furthermore, retrotransposon LTR sequences were shown to induce natural RNAi due to RNA duplex formation (Sijen and Plasterk 2003). The RNAi machinery has been implicated in gene silencing in two independent modalities: at the RNA level through degradation of mRNAs and at the chromatin structure level through induction of methylation (Dykxhoorn et al. 2003; Ekwall 2004). Both silencing pathways might be affected by editing of repetitive-element foldback structures. Silencing of RNAs containing such inverted repeats might be prevented through their modification by RNA editing and their subsequent nuclear retention (Zhang and Carmichael 2001) or by rendering those RNAs inadequate substrates of the RNAi machinery. It is possible that the observed embryonic lethality and apoptosis in A-to-I editing-deficient mice (Wang et al. 2000, 2004; Hartner et al. 2004) is related to the breakdown of this control mechanism leading to the posttranscriptional silencing of essential genes.

The work presented here has been based on the analysis of cellular mRNAs that contain Alu repeat elements. However, the underlying principles probably also apply to Alu RNAs generated from transcriptionally active Alu elements. Alu elements do not encode transcription termination signals (Deininger 1989), and thus read-through transcription from transposition-competent Alu repeats can result in intramolecular Alu pairs, leading to the editing of a sequence that subsequently becomes retrotranscribed. Editing of primary transcripts of repetitive elements may have an important role in the control of their proliferation and a dedicated analysis of such transcripts for editing events represents an important future direction.

A recent study by Levanon et al. (2004) reported a computational approach for the identification of heavily edited genes in the human transcriptome and found that editing mostly occurs in Alu repeat elements (greater than 92% of the substrates identified), giving us the opportunity to compare the two approaches and datasets. The computational strategy used by Levanon et al. (2004) differs substantially from ours both in the sequence dataset employed and in the methodology applied. The use of expressed sequence tags (ESTs; in contrast to our use of mRNA sequences) offers a much larger primary dataset for analysis; however, single-pass sequences have a higher error rate (Liang et al. 2000), and EST databases are biased toward sequences near 3′-termini of mRNAs (Liang et al. 2000). Levanon et al. (2004) selected candidate sequences for editing by identifying inverted repeats followed by the evaluation of AtoG mismatch rates, whereas we directly evaluated the AtoG mismatch content in repetitive elements irrespective of the presence of a nearby pairing sequence. The approach of Levanon et al. (2004) allows the discovery of edited inverted repeats that do not belong to any of the repetitive-element families (although the previously known brain substrates were missed), but it does not identify cases where a base-pairing sequence is not evident because of truncated cDNA and EST sequences and incomplete knowledge of gene boundaries. We found that for approximately one-third of the edited Alu elements a pairing Alu cannot be located within the gene boundaries as determined by known mRNAs, although in most of the cases it can be identified at the genome level. A comparison of the edited gene/mRNA datasets of the two studies shows a 34.5% overlap when gene names and symbols are compared. It should be noted, though, that editing of the same gene might reflect editing at different sites or within different Alu elements of the same gene.

The two approaches are overlapping as well as complementary. Taken together, they have probably uncovered the most significant part of the heavily edited exonic sequences for which sequence data are available. From our analysis we estimate an additional approximately 4,000 edited Alu elements besides the 1,925 Alus that we have selected as a very high confidence set. Thus, it is important to note that the heavily edited sequences represent the tip of an iceberg with many more mRNAs in the human transcriptome being edited at single or a small number of positions.

## Materials and Methods

### 

#### RNA editing analysis

Human brain samples were provided by the Harvard Brain Tissue Resource Center, Belmont, Massachusetts, United States; human lung cDNA was from Clontech (Palo Alto, California, United States). Total RNA isolation and reverse transcription have been described previously (Ausubel et al. 1995; Maas et al. 2001). Gene-specific PCR was performed as described earlier (Maas et al. 2001), and a list of oligonucleotide primer sequences used in this study is available on request. RNA editing analysis was done by direct sequencing of gene-specific, gel-purified RT-PCR products as described (Maas et al. 2001), using an automated ABI310 (Applied Biosystems, Foster City, California, United States) capillary electrophoresis sequencer. Human gDNA used for gene-specific PCR was isolated from the same tissues according to standard protocols (Ausubel et al. 1995).

#### Computational procedures

For analysis of the pool of human cDNA sequences we developed a program named Procedures for Repetitive Element Foldback Analysis (PREFA). We used the set of cDNA sequences from the UCSC database (July 2003) comprising 103,723 sequences (after removal of duplicate entries). The set of repetitive elements (for Alus 1,163,041 unique elements) and related information of the human genome (created with RepeatMasker based on the Repbase [Jurka 2000] release of June 2002) was obtained from the same source. For each examined repetitive-element family we first selected the subset overlapping partially or fully with genes. For Alus the number is 2,003,976, including duplicates, or 572,107 unique sequences. From this subset we then selected those overlapping with exons (31,666).

The RNA and genomic sequence for each element was extracted and compared base by base for mismatches. A small number of cases with very high non-AtoG mismatches (greater than 20/element) were discarded as misaligned or erroneous. From the repetitive elements showing at least a single AtoG change we selected those where mismatch distribution cannot be accounted for by SNPs and sequence errors using the following procedure:

The overall expected ratio of AtoG discrepancies relative to the total number of mismatches was calculated from the whole sample, assuming the expected AtoG mismatches to be approximately equal to the average of the rest of the transitions:



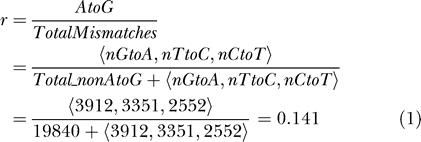



The expected probability for an AtoG mismatch at a single position in a given element was calculated from the total number of mismatches found in the element in cases where other mismatches were present (2) or from the whole sample where only AtoG mismatches were found (3): Here nAtoG and nOther is the total number of AtoG and non-AtoG mismatches found for this element:



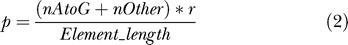





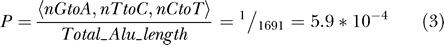



Given the probability *p* for an AtoG mismatch to occur at any given position, the expected values for the number of AtoG were calculated:



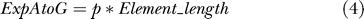



A χ^2^ test was calculated for each element and those with a χ^2^ value exceeding the critical value (for α = 0.000001) were selected as edited, and these values correspond to approximately more than five AtoG changes in the absence of any other change in the approximately 300 bp of an Alu).

For each element in the high-confidence set the closest inverted element was identified among the elements present in the same gene boundaries. The distance separating the pair was calculated from the location of the first base of each element, according to the genomic sequence numbering and irrespective of their orientation. The divergence of each element was derived from the corresponding entry in the UCSC annotation database (ChrN_rmsk) representing mismatches per hundred bases. Tissue of origin of the RNAs was also derived from the UCSC mRNA annotation. For RNAs described to originate from multiple tissues, the corresponding RNAs were included in the count for each of those tissues. RNAs originating from a specific subregion of a tissue, such as subareas of the brain, were counted within the subregion but not in the whole-tissue set of RNAs.

Alignment of the Chromosome 1-derived Alu sequences was performed with the MegAlign program of the DNASTAR (Madison, Wisconsin, United States) package (Lasergene) using the CLUSTAL algorithm (Jeanmougin et al. 1998). Further manual adjustments were necessary owing to the presence of simple repeats in Alu sequences. Analysis of the alignment and base counts surrounding the editing sites were done with PREFA.

## Supporting Information

Table S1 Database of Computationally Identified Editing Targets The database lists the GenBank accession numbers, gene names, gene product description, chromosome location, and type of Alu element and location within the mRNA sequence, the identity of the most likely pairing Alu elements within the same gene, and the distance in base pairs (bp) between the pairing Alus. The positions of all predicted editing sites within the individual sequences can be viewed by pasting the accession number into the USCS genome browser (Kent et al. 2002) at http://genome.ucsc.edu/cgi-bin/hgGateway and following the link to mRNA/Genomic alignment. We found that six cDNAs map on two chromosomes (AB095924, AK021666, AK055562, AK092837, AK094425, and BC039501); details are given for the most plausible assignment. We have observed that in the 43 cases that we experimentally analyzed, usually additional editing sites were identified when directly sequencing gene-specific PCR products.
(276 KB XLS).

### Accession Numbers

The GenBank ((http://www.ncbi.nlm.nih.gov/Genbank) accession numbers for the genetic sequences discussed in this paper are LUSTR (AB046844), KIAA0500 (AB007969), BTKI (AB037838), KIAA1497 (AB040930), and GPR81 (BC0067484).

The Entrez Gene (http://www.ncbi.nlm.nih.gov/entrez/query.fcgi?db=gene) ID numbers for ADAR1, p53, SIRT2, NFκB, and SPG7 are 103, 7157, 22933, 4790, and 6687, respectively.

## Abbreviations

ADAR: adenosine deaminase acting on RNA
BTKI: Bruton's tyrosine kinase
dsRNA: double-stranded RNA
EST: expressed sequence tag
gDNA: genomic DNA
HUGE: Human Unidentified Gene-Encoded
PREFA: Procedures for Repetitive Element Foldback Analysis
RNAi: RNA interference
siRNA: small interfering RNA
SNP: single-nucleotide polymorphism
